# Effect of Freeze–Thaw Cycles (FTCs) on the Mechanical Behavior of Highway Clay Subgrade Soils Stabilized with Lime and Polypropylene Fibers

**DOI:** 10.3390/polym17172405

**Published:** 2025-09-04

**Authors:** Tayfun Şengül, Yaşar Vitoşoğlu

**Affiliations:** Department of Civil Engineering, Faculty of Engineering, Kütahya Dumlupınar University, 43100 Kütahya, Türkiye; yasar.vitosoglu@dpu.edu.tr

**Keywords:** soils stabilization, lime, polypropylene fiber, freeze–thaw cycles, shear strength

## Abstract

High-plasticity soils pose significant problems in road infrastructure, adversely affecting structural safety due to their unfavorable engineering properties. Lime stabilization is one of the most widely used methods for improving such soils. However, lime addition may cause brittleness of these soils, resulting in a sudden loss of strength. To overcome this weakness, this study investigated using polypropylene fibers in combination with lime stabilization. Accordingly, the plasticity, compressibility, and strength properties of soil mixtures containing 3%, 6%, 9%, and 12% lime, along with mixtures prepared with a constant 0.5% polypropylene fiber content, were systematically evaluated in a laboratory environment. Additionally, the influence of freeze–thaw cycles (FTCs), which induce long-term strength degradation in highway subgrades, on these mixtures was investigated. The results indicated that lime addition reduced the plasticity index by up to 38% without causing a significant change in dry unit weight. It was also determined that FTCs could lead to a strength loss of up to 84% in natural soil, and this loss was substantially reduced by adding lime and fibers. These findings highlight that the lime-fiber combination represents an effective and sustainable method for increasing the performance of high-plasticity soils.

## 1. Introduction

The environmental impact of road construction on ecosystems is harmful, mainly because it requires large amounts of mineral materials to construct granular base and sub-base layers and create suitable subgrades. Since weak subgrades require making highway pavement layers thicker, it increases pavement construction costs and the negative impact of road construction on the natural environment. When the characteristics of natural subgrades are not suitable for road construction, suitable materials need to be obtained from nearby rivers or surrounding borrowing areas. Instead of using borrowed materials, improving these subgrades to meet the desired performance criteria can be more economical. Furthermore, improving existing subgrades minimizes environmental impacts by reducing the need for excavation and backfilling.

Improving highway subgrades involves rehabilitating existing soil properties through physical and/or chemical methods to increase the road infrastructure’s bearing capacity, durability, and longevity. This process aims to make soils with weak or insufficient bearing capacity suitable for road construction.

The subgrade is the lowest layer that the highway pavement directly contacts and it carries the load. Due to a weak subgrade, collapses, cracks, deformations, and deterioration of the road surface may occur over time. Therefore, soil improvement is carried out to increase the soil’s bearing capacity, reduce volume changes, and enhance its resistance to water. The soil improvement method to be applied depends on the soil type, moisture status, loading conditions, and economic criteria. The methods used in soil improvement can be grouped under two headings: mechanical and chemical improvement methods. These two methods can be used independently or in combination to improve the properties of a subgrade from various perspectives.

Mechanical improvement methods are processes in which the physical properties of the soil are modified without changing its chemistry. In these methods, soil improvement is achieved by changing the physical structure of natural soil particles through vibration or compaction, or by adding other objects such as barriers and nails to the soil structure. Mechanical improvement methods also involve mixing and compacting the subgrade with other materials of varying grain sizes in specific proportions, resulting in a final mixture with the targeted grain distribution. Finally, reinforcing the soil using synthetic or natural additives to improve the properties of the subgrade is also one of the mechanical improvement methods. Adding randomly distributed fibers with high tensile strength to the soil prevents erosion and settlement and increases the shear strength of the subgrade.

Chemical improvement or stabilization is achieved by changing the chemical structure of the soil by adding various additives, such as fly ash, lime, cement, or polymer, to the subgrade. These substances react chemically with the soil and increase its bearing capacity, water resistance, and durability. In addition, the purpose of chemical stabilization of soils is to ensure cementation by increasing the size of the grains in the soil material, reducing the plasticity index and swelling-shrinkage potential, and increasing their stability. Chemical stabilization of soils is a practical, long-lasting, and permanent method. It is often cost effective because the amount of chemical additives required for effective stabilization is small. This method is also compatible with most soil types and allows the reuse of industrial by-products as chemical additives.

Lime is one of the most commonly used additives in highway pavements. Because it is economical and straightforward to apply, it is widely used in foundations, embankments, and other soil structures [1]. Lime stabilization provides effective results, especially in high-plasticity clays that contain enough silica and alumina to react chemically with lime. Additionally, clayey gravels and silty clays react readily with lime. On the other hand, lime stabilization will not yield the desired results in granular, low-cohesion, or cohesionless soils without the addition of pozzolan.

Some studies have reported that lime does not significantly affect compaction parameters. However, lime undergoes a rapid and extensive chemical reaction with soil particles compared to other binders. Changing the characteristics of the soil through chemical interaction improves the soil’s various properties, such as compression and strength parameters [2,3,4].

Within the scope of lime stabilization, Kavak and Akyarlı (2007) [2] evaluated the improvement obtained due to lime stabilization applied to a road section dominated by green and brown clays. Adding 5% lime to both clays achieved lime stabilization under field conditions. California bearing ratio (CBR) tests in the laboratory showed increases of up to 16 and 21 times in green and brown clays, respectively, compared to initial CBR values after 28 days.

In some studies, sewage sludge ash was used in addition to lime to stabilize cohesive soils. In such a study conducted by Lin et al. (2007) [5], five different ratios of sludge ash to slaked lime, 0%, 2%, 4%, 8%, and 16% by weight, were mixed with cohesive soil, and their effects on the soil were investigated. The experimental findings revealed that the unconfined compressive strength (UCS) of samples containing additives was enhanced three to seven times compared to untreated soil. The triaxial compression test results also showed that cohesion increased with increasing additive amount.

Sobhan and Mashnad (2002) [6] reported that chemical stabilization significantly increased the compressive strength of the soil but made little contribution to the tensile strength. This issue becomes a significant problem when a shrinkage crack occurs in soil. Accordingly, improving the tensile strength, ductility, and toughness of lime-treated soil through fiber reinforcement becomes essential.

In this context, synthetic and natural fibers are widely used to improve subgrades [7,8]. Mohammed et al. (2022) [9] evaluated the strength properties of a problematic soil by reinforcing the soil sample with sisal fibers. To obtain and assess various parameters, samples containing sisal fibers at 0.5%, 1%, 1.5%, and 2% by dry weight of the soil and with fiber lengths of 10, 15, and 20 mm were prepared and subjected to compression, unconfined compressive strength (UCS), and soaked CBR tests. The study demonstrated that increasing the fiber content and length caused a slight reduction in the maximum dry density of the reinforced soil. On the other hand, the optimum moisture content showed a moderate increase. In addition, notable increases in the UCS and CBR were observed as the fiber length and content were increased.

Synthetic fibers have been used to reinforce soils for nearly forty years. Polypropylene fibers, which are utilized to improve soil strength and reduce shrinkage, are the most commonly used additive in soil reinforcement. According to Puppala and Musenda (2000) [10], reinforcing soil with polypropylene fibers enhances the soil’s UCS and decreases the volumetric shrinkage strain and swelling pressure in expansive clays. In another study conducted by Abd Al-Kaream et al. (2022) [11], experiments were performed on clay soil containing polypropylene fibers in amounts ranging from 0.5% to 1.5%, and it was observed that polypropylene fibers increased the liquid and plastic limits, as well as the UCS. The compression index decreased by 69% and the swelling index decreased by 78%. As the percentage of polypropylene fibers increased, the specific gravity and maximum dry unit weight decreased.

Apart from polypropylene fibers, synthetic fibers made from other materials, such as polyester, polyethylene, glass, and nylon, are also used to reinforce floors [12,13]. Kumar et al. (2006) [14] conducted UCS tests on clay samples reinforced with straight and crimped polyester fibers at fiber contents of 0%, 0.5%, 1.0%, 1.5%, and 2.0%. The study’s findings indicated that the UCS increased with increasing fiber length and/or fiber content.

A review of existing studies on fiber-reinforced lime-treated soils indicates that incorporating fibers can mitigate the brittle fracture typically encountered with lime stabilization [15,16,17,18]. In this context, Cai et al. (2006) [15] carried out a study to investigate the influence of lime and polypropylene fibers on the engineering characteristics of soft soil. The study demonstrated that adding fibers to lime-stabilized soil enhanced its UCS and shear strength parameters. Consoli et al. (2012) [16] indicated that porosity, fiber content, and lime content are effective variables for improving lime and fiber-stabilized soil’s compressive and tensile strength.

One crucial issue to consider in pavement design is the behavior of stabilized clay soils under freeze–thaw conditions. Freezing and thawing cycles weaken the soil structure and reduce the performance of pavement materials, causing microcracks in compacted soils. It is known that pavements exposed to frost effects have shorter service lives compared to those not exposed to the same impact [19].

Guney et al. (2006) [20] emphasized that the stable material obtained must resist additional stresses arising from freeze–thaw cycles (FTCs) in every application related to soil stabilization.

Hotineanua et al. (2015) [21] studied the effect of FTCs on the mechanical properties of two types of plastic soils stabilized with lime. Their research investigated two types of clay soils, low-plasticity kaolinite and high-plasticity bentonite, under untreated and lime-stabilized states for curing durations of up to 300 days. The findings indicated that the volume of stabilized soils increased during early FTCs, but this growth became less significant in subsequent cycles. The UCS increased significantly as the curing period was prolonged from 3 to 28 days, up to 300 days. Following exposure to freeze–thaw conditions, cracks caused by ice lens formation within the pores of lime-stabilized soil samples were found to have a greater effect on bentonite soil than kaolinite soil.

There are limited studies on the freeze–thaw behavior of fiber-reinforced soils [22]. In this regard, Liu et al. (2020) [23] examined the impact of FTCs on the strength of soil reinforced with natural fibers. For this purpose, single fiber tensile tests, unconfined compression tests, and scanning electron microscopy (SEM) analyses were conducted on soil reinforced with cotton straw fibers subjected to 0, 3, 5, 10, 15, and 20 freezing and thawing cycles. The UCS of the soil reinforced with fibers declined exponentially as the number of FTCs increased. The reduction in UCS of fiber-reinforced soil under freeze–thaw conditions was smaller than the strength loss occurring at the fiber–soil interface, as reinforcement performance depends both on fiber–soil interaction and the spatial stress network generated by the fibers. SEM observations also confirmed the existence of an intricate spatial stress system, which enhances fiber reinforcement after FTCs. In another study, Ardakani and Aliaghaei (2025) [24] investigated the effectiveness of adding carbon fibers to clay soil to improve its performance during FTCs. Carbon fiber was mixed with clay at proportions ranging from 0.1% to 0.4% by weight of dry soil. Dynamic tests were performed on unreinforced and reinforced samples after exposure to 0, 3, 6, and 9 FTCs under 100 kPa and 300 kPa confining pressures. The findings revealed that adding carbon fibers increased both the shear modulus and damping ratio.

Boz and Sezer (2018) [25] conducted a comparative investigation into the effects of FTCs on lime-stabilized clay soil reinforced with polypropylene and basalt fibers. Soil specimens reinforced with 12 mm long polypropylene and basalt fibers at weight fractions of 0%, 0.5%, and 1% were compacted utilizing standard Proctor compaction energy, applied at the corresponding optimum moisture contents. For comparison, certain fiber-clay mixtures were stabilized with 3% lime, while others remained unstabilized. All samples were cured for 28 days in a humid chamber at an average temperature of 20 °C, after which they were subjected to freeze–thaw testing for 0, 1, 3, 7, and 10 cycles. A series of unconfined compression and ultrasonic pulse velocity tests was conducted, demonstrating that an increase in FTCs decreased the UCS and ultrasonic pulse velocity across all tested samples. Additionally, mass loss was significant in samples subjected to freeze–thaw cycles. Polypropylene fibers were more effective against freeze–thaw cycles than basalt fibers in terms of freeze–thaw resistance.

In this study, changes in the engineering properties of soils stabilized with different proportions of lime (3%, 6%, 9%, and 12%) and a constant proportion of polypropylene fibers (0.5%) were experimentally investigated for the improvement of high-plasticity highway subgrades. In this context, the stabilized soil sample properties, such as plasticity, compressibility, and shear strength, were evaluated. Furthermore, in this study, where lime and polypropylene fiber additives were used together, the effects of different curing times (uncured, 1, and 28 days) and 28-day FTCs consisting of 12 h cycles on the UCS of the soils were investigated in detail. Based on the findings, the optimum lime additive ratio that would maintain long-term resistance against FTCs was determined for both fibrous and fiberless conditions, and recommendations were presented to enhance the sustainability of road pavement performance.

## 2. Materials

### 2.1. High-Plasticity Subgrade Soil

The subgrade used in this study was a high-plasticity clay soil obtained locally from Kütahya. The natural water content of the subgrade soil (w) was around 22%, which varied depending on seasonal differences. Soil samples were obtained 1–2 m below the surface and dried in an oven at 105 °C before being blended and homogenized. Sieve and hydrometer analysis, Atterberg limits, and specific gravity tests were carried out to determine the physical characteristics of the natural soil, and the results are shown in Table 1. The specific gravity of the natural soil was 2.7. Based on the American Association of State Highway and Transportation Officials (AASHTO) soil classification system, it was classified as an A-7-5 clayey soil, which is generally regarded as having moderate to poor suitability for road infrastructure applications. According to the Unified Soil Classification System (USCS), the soil belonged to the MH group, comprising high-plasticity inorganic silts and clayey silts.

**Figure 1:**
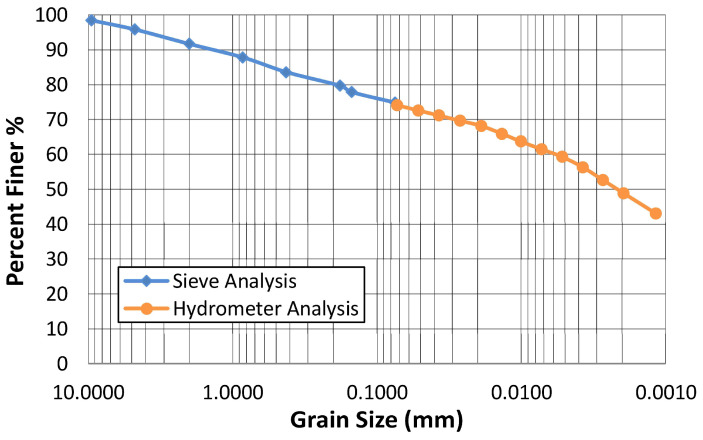
Grain size distribution curve of natural soil.

To assess the grain size distribution of the natural soil, the wet analysis method specified in ASTM D1140-00 (2006) [26] was employed to separate fine-grained soils smaller than 75 µm (No. 200 sieve) from coarse-grained soils. The grain size distribution curve determined by sieve analysis according to ASTM D6913-04 (2009) [27] and hydrometer analysis according to ASTM D7928 (2016) [28] is shown in Figure 1.

**Figure 2:**
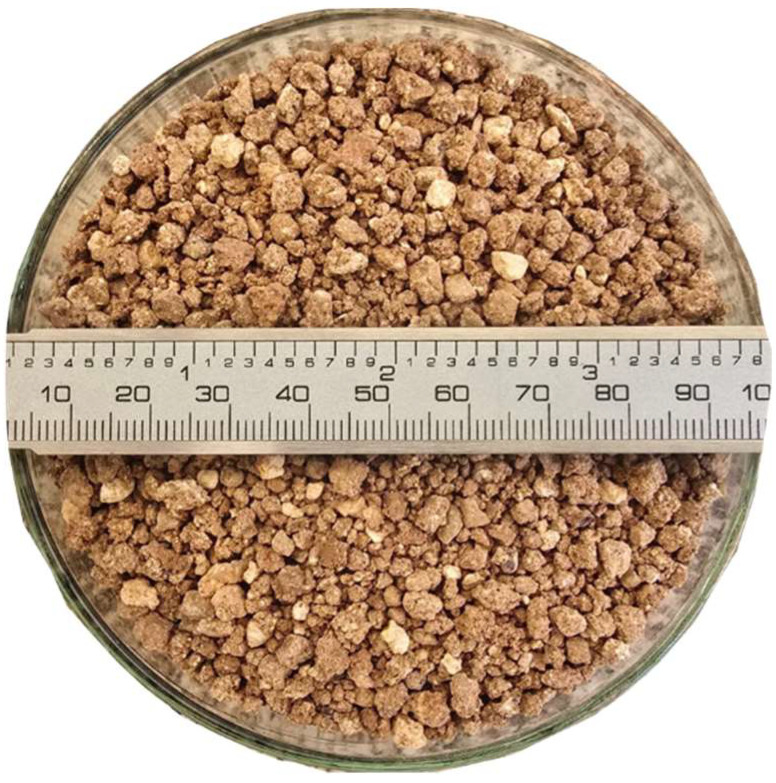
Photograph of natural soil (<4.75 mm).

**Figure 3:**
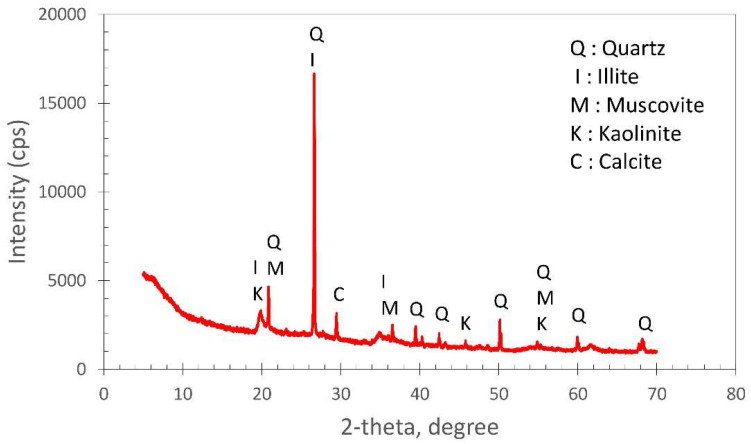
The XRD pattern of subgrade soil.

According to the results, the natural soil consisted of 4.2% gravel, 21% sand, and 74.8% fine-grained soil. A photograph of the natural soil is shown in Figure 2, and the average diameter of the grains forming the soil was 0.0022 mm, which was clay sized. The elemental composition of the natural soil, as determined through X-ray fluorescence (XRF) spectroscopy (PANalytical/Axios Max. It is produced in the Netherlands), is presented in Table 2. In the chemical analysis determined by XRF spectroscopy, the loss on ignition was 12.01%. In addition, X-ray diffractogram (XRD) analysis of the natural soil was carried out using a PANalytical Empyrean (The Netherlands) employing Cu Kα radiation (λ = 1.54 Å) over a 2θ range of 5–70°. The obtained XRD patterns of the soil are illustrated in Figure 3.

According to the XRF results, the most abundant principal oxides were found to be 54.72% SiO_2_, 15.27% Al_2_O_3_, 8.01% F_2_O_3_, 4.12% CaO, and 2.76% MgO.

This XRD pattern was similar to the typical diffraction pattern of clay samples, and the high quartz content and clay phases were notable. It was observed that the subgrade consisted of quartz (SiO_2_), illite ((KH_3_O)(Al,Mg,Fe)_2_(SiAl)_4_O_10_[(OH)_2_(H_2_O)]), muscovite (KAl_2_(Si_3_Al)O_10_(OH,F)_2_), kaolinite (Al_2_Si_2_O_5_(OH)_4_), and calcite (CaCO_3_) [29]. In the XRD analysis, sharp peaks on the 2-theta axis indicated certain crystal structures. The very sharp peak at 26.6° indicated the quartz phase, and this result was confirmed by 54.72% SiO_2_, the most dominant oxide in the XRF analysis. In the XRF analysis, %15.27 Al_2_O_3_ + %1.59 K_2_O + %2.76 MgO indicated clay minerals. This result, along with small peaks around 19–25° and 35° in the XRD analysis, confirmed the presence of clay minerals such as illite, kaolinite, and muscovite. Despite the high iron content of 8.01% in the XRF analysis, no distinct hematite (Fe_2_O_3_) peak was visible in the XRD analysis. This result suggested that the iron may be amorphous or dispersed within the clay structure.

Furthermore, the peak identified at 29.4° in the XRD analysis indicated the presence of calcite and was consistent with the 4.12% CaO content determined in the XRF analysis. CaO and MgO also indicated the presence of minerals with potential for stabilization and reactivity. Overall, the XRF and XRD results revealed a consistent material profile.

**Figure 4:**
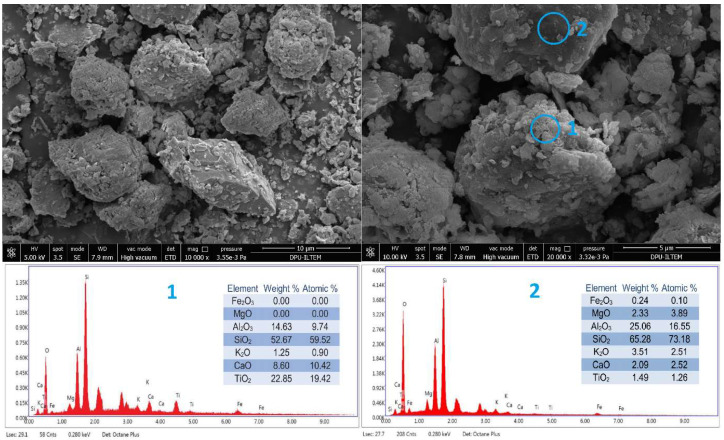
Surface morphology and chemical composition of natural soil by SEM-EDX analysis.

The surface morphology of the natural soil was examined using scanning electron microscopy (SEM), while its chemical composition was characterized through energy-dispersive X-ray spectroscopy (SEM-EDX). The results are presented in Figure 4.

The SEM analysis of the natural soil showed that particles of varying sizes and shapes had an irregular structure. X-ray spectrometry (EDX) (The brand of Scanning Electron Microscopy (SEM) device is FEI Nova NanoSEM 650. It is produced in the Netherlands by the PHILIPS / FEI company) showed that the particles’ main composition in spectrum 1 consisted of SiO_2_, TiO_2_, Al_2_O_3_, and CaO. The significant difference between the high TiO_2_ content detected in Spectrum 1 and the overall chemical composition presented in Table 2 was thought to be due to the heterogeneous phase distribution of the sample. Spectrum 1 represented a local TiO_2_-rich phase at the microstructural level analyzed using the EDS method. By contrast, the 0.83% TiO_2_ value in Table 2 reflected the average result of the overall composition of the sample. In multiphase materials, local regions with high TiO_2_ content may exist, but these regions do not significantly affect the macroscale chemical composition of the sample. Therefore, differences can be observed between the results of pointwise EDS analyses and the overall chemical analysis.

It was seen that the main composition of the particle in Spectrum 2 consisted of SiO_2_ and Al_2_O_3_ (Figure 4). Therefore, it was confirmed that it was consistent with the data obtained from the chemical analysis results (Table 2), and its main composition consisted mainly of SiO_2_ and Al_2_O_3_.

### 2.2. Lime

**Figure 5:**
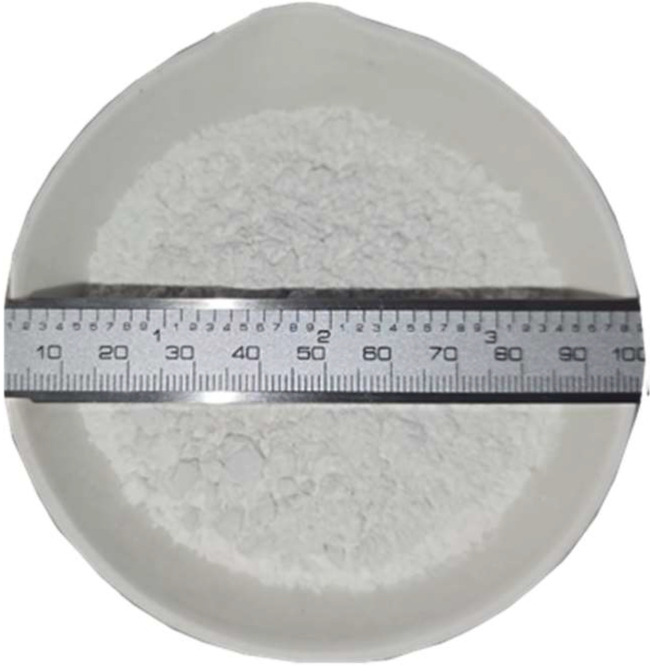
Photograph of slaked lime.

Slaked lime (calcium hydroxide) is produced by water hydrating quicklime (calcium oxide) with water. It represents a practical and efficient material that can be applied in liquid and powder forms for various purposes. The slaked lime used in the study was commercially available in powder form and produced by the Adacay company. A photograph of the slaked lime is shown in Figure 5, and its physical and chemical properties are given in Table 3. It typically comprises a minimum of 90% calcium oxide (CaO) and up to 2% magnesium oxide (MgO). In this study, 100% of the lime passed through a 0.15 mm sieve diameter and 93.5% passed through a 0.075 mm sieve diameter. To reduce the carbonation effect caused by moisture, the lime was kept in an airtight plastic container.

### 2.3. Polypropylene Fibers

**Figure 6:**
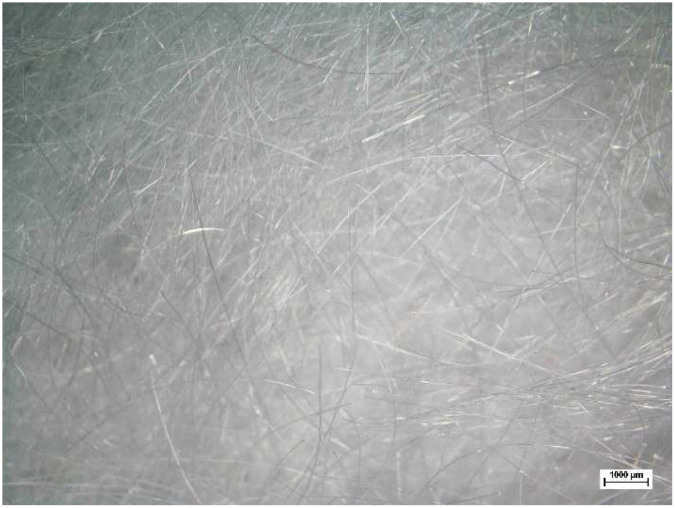
Photograph of 12 mm long polypropylene fibers.

For this study, commercially available synthetic plastic fibers in the form of polypropylene, designated as BF-12 and produced under the Betonfiber brand, were also utilized. Polypropylene fibers are preferred synthetic fibers because they are a low-cost, non-toxic, hydrophobic, and chemically inactive material. They also have high corrosion, acid, and alkali resistance and high tensile strength. Commercially available polypropylene fibers have the characteristics of being fine and fibrous and they are found in clusters. As specified by the manufacturer, the technical characteristics of the polypropylene fibers utilized in this study are listed in Table 4, and a photograph is shown in Figure 6. The specific gravity of the fibers was 0.91 g/cm^3^. Their diameters ranged from 18 µm to 20 µm, and their length was 12 mm. Their tensile strength and modulus of elasticity were 450–700 N/mm^2^ and 3000–3500 N/mm^2^, respectively.

## 3. Results and Discussion

To stabilize the subgrade soil with lime, lime was mixed homogeneously with the soil in dry form at the rates of 3, 6, 9 and 12% by dry weight, and the plasticity properties of these soil and lime mixtures were determined. Compaction tests were conducted to determine the optimum moisture content and maximum dry unit weight values that yielded the most effective compaction for both the natural soil and lime-added soil mixtures. Unconfined compression tests were also performed to evaluate the variation in shear strength of the prepared samples by applying standard compression energy at optimum water content with mixtures with determined compaction characteristics.

### 3.1. Plasticity Properties

According to ASTM D4318-05 (2008) [30], the consistency limits of the soil-lime mixtures were determined, as summarized in Table 5.

In the improvement of the natural soil with lime, the addition of lime at rates varying between 3% and 12% caused decreases in the liquid limit value (*W*L) of the soil between 4.7% and 25.0% and in the plastic limit (PL) value between 4.4% and 13.0%. It was determined that increasing the amount of lime in chemical stabilization caused a decrease in the plasticity index (PI) between 4.9% and 38.1%.

The plasticity index of a soil is a key parameter that is directly correlated with its swelling pressure and swelling potential. Since increasing the amount of lime in chemical stabilization causes a decrease in the plasticity index, the swelling pressure is expected to decrease [2,3,31].

### 3.2. Compaction Behavior

**Figure 7:**
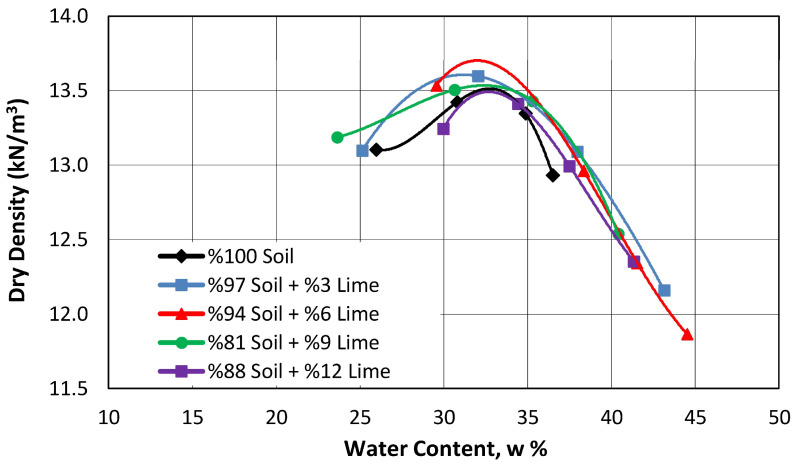
The impact of incorporating lime at various proportions on the compaction properties of the soil.

The compaction characteristics of the natural soil incorporating varying proportions of lime were determined by ASTM D698-07 [32], and the corresponding results are presented in Figure 7.

In general, many researchers have reported that adding lime to soils causes an increase in the optimum water content and a decrease in the maximum dry density [3,4,33].

When the maximum dry unit volume weight and optimum water content values obtained for the mixtures in this study were compared with those of the natural soil, it was seen that increasing the lime content of the mixture up to 6% caused an increase of approximately 1.4% in the dry unit volume weight values and a decrease of 3% in the optimum water content values. The increase in dry unit weight could be attributed to the relatively finer particle size of lime compared to the natural soil, which reduced the void ratio by occupying the pore spaces within the soil matrix. At the same time, since the lime additive increased the workability of the soil, it could be compacted at lower water contents.

When the lime content of the mixture was increased from 6% to 12%, a decrease in the dry unit weight and an increase in the optimum moisture content were observed, reaching the levels obtained for the natural soil. If the lime concentration in the mixture exceeded the optimum level, the dry unit weight value decreased because lime particles with relatively low specific gravity replaced a particular volume of soil particles. At the same time, lime caused particles to clump and occupy larger volumes due to the cation exchange reaction, which changed the compaction characteristics of the soil. The increase in the optimum water content due to increased lime content was likely due to the high water retention of lime.

### 3.3. Shear Strength

**Figure 8:**
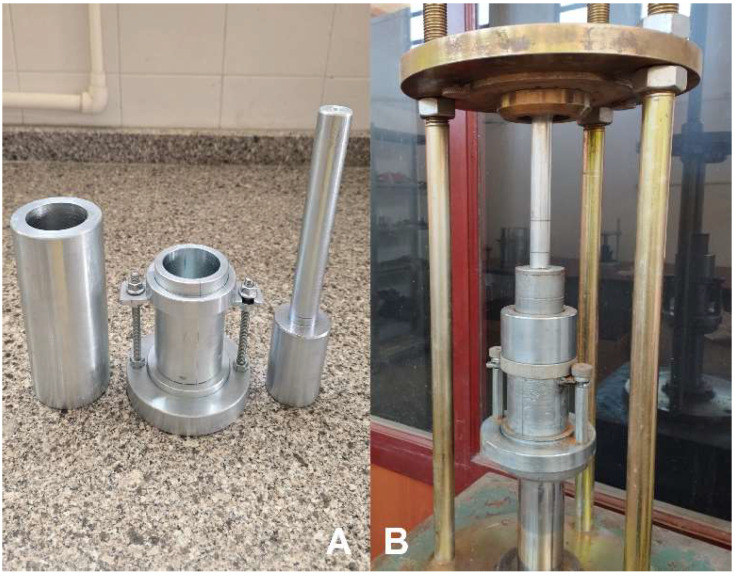
Preparation of unconfined compression samples using the static compression method. (**A**) Sample preparation mold and piston; (**B**) Compression of the sample.

**Figure 9:**
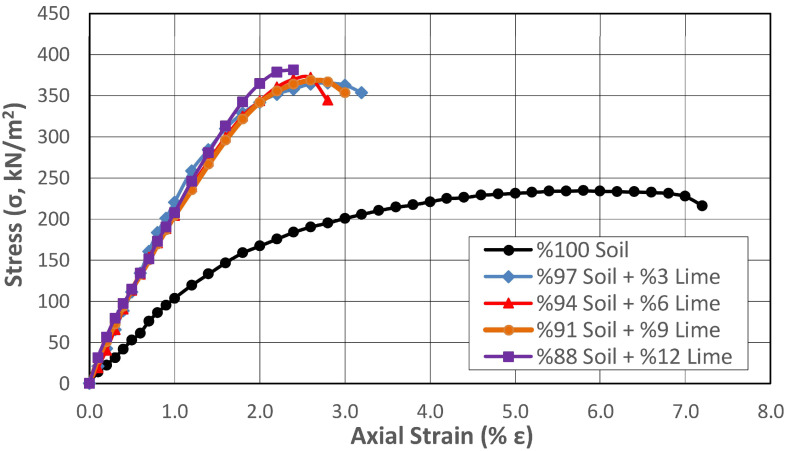
Change in shear strength of lime-stabilized subgrade at different mixing ratios.

The natural soil and its lime-stabilized mixtures were prepared at their respective optimum moisture contents, which were determined individually through compaction tests. Then, the samples were placed in a stainless steel mold with a diameter of 50 mm (Figure 8A) and compressed statically with the help of a piston pushed at a constant speed with the help of a hydraulic jack (Figure 8B) to obtain the samples for which the shear strength parameters would be determined. The preparation of unconfined compression samples by the static compression method is shown in Figure 8. In the standard compaction method, when a sample is compressed in three layers, the formation of shear planes between the layers can affect the strength of each sample at different levels. Therefore, the static compression method was adopted in this study. In the static compaction method, the wet soil mass with the optimum water content that will fill the sample volume of standard dimensions after compaction is determined and the sample is placed in the compaction mold. Thus, by applying standard compaction energy in the compaction test, samples with the same void ratio and dry unit volume weight as the compressed sample can be obtained. Unconfined compression tests were conducted on the prepared soil samples following ASTM D2166-06 [34], and their strength characteristics were subsequently evaluated. The changes in shear strength of the subgrade soil stabilized with different proportions of lime are shown in Figure 9.

**Figure 10:**
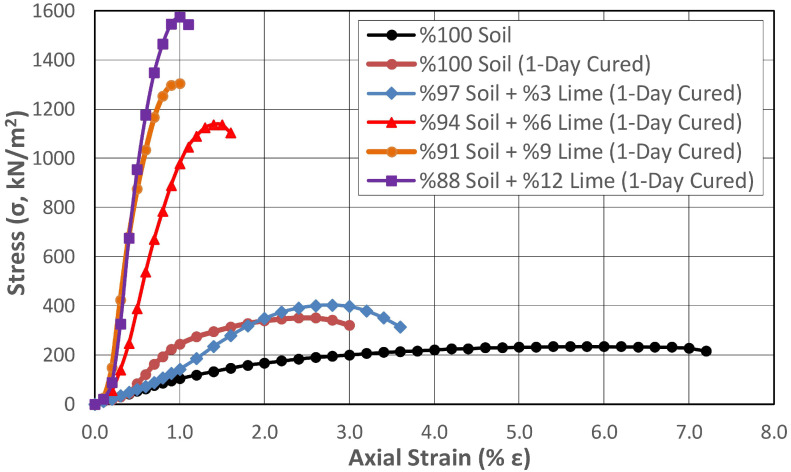
Change in the shear strength of the lime-stabilized subgrade at the end of 1 day of curing with different mixing ratios.

**Figure 11:**
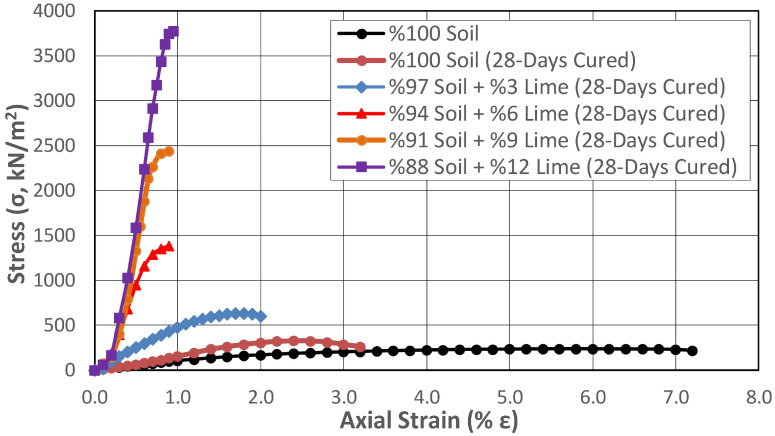
Change in shear strength of the lime-stabilized subgrade at different mixing ratios after 28 days of curing.

The change in the UCS of soil samples stabilized with lime addition was determined by gradually increasing the lime addition at rates between 3% and 12% with 0, 1, and 28 days of curing. When the strength properties of the lime-added samples were examined immediately after preparation without being exposed to curing conditions (Figure 9), it was determined that gradually increasing the lime addition at rates varying between 3% and 12% increased the strength at rates ranging between 55.7% and 62.6%. According to the results obtained, although the strength properties increased depending on the increase in the lime percentage of the mixture, the difference in strength between the lowest and highest lime percentage was 4.5%. This result showed that obtaining sufficient efficiency from the additive in the chemical stabilization method with lime depended on completing pozzolanic reactions between the lime and the soil and providing appropriate curing conditions and sufficient time for these to occur. For this purpose, samples containing lime were prepared at the same mixing ratios and stored under ideal curing conditions at constant room temperature (20 °C) for 1 and 28 days without changing the water content to determine the UCS. The results obtained are presented in Figure 10 and Figure 11, respectively.

When the results were examined, it was determined that the strength increased by approximately 50% in the case of only one day of curing, even when no additives were added to the subgrade. It was thought that the most important reason for this increase was due to the pozzolanic reactions that occurred when the 4.12% quicklime (CaO) in the natural soil absorbed the moisture in the environment under curing conditions. When the strength of the one-day cured sample without an additive and the sample with 3% lime additive were compared, it was determined that there was a 15% increase, and when the lime additive contents were 6% and 12%, these increases were 224% and 349%, respectively.

It was observed that the differences between the shear strengths of the 28-day cured samples with different amounts of lime added increased significantly. While the shear strength of the 3% added sample increased by 94%, the shear strength of the 12% added sample increased by 1063%.

When the curing effect was examined based on the changing lime percentage, it was determined that the shear strength obtained in the 28-day cured condition in samples containing 3% lime increased by 73% compared to the uncured condition. These increases were 272% and 561% at increasing lime mixture ratios (6% and 9%), while it reached 891% at the highest lime mixture ratio of 12%.

Kavak and Baykal (2012) [35] investigated the changes in the microstructure of kaolinite cured for a long time and stabilized with lime. Unconfined compression test samples with 4% and 12% lime content by weight were prepared and hardened in a humidity chamber for curing. Firstly, the UCS of pure kaolinite was found to be 125 kPa. This value increased to 1015 kPa after one month and 2640 kPa after 10 months for the cured and lime-stabilized samples. Similar strength increases were observed for kaolinite stabilized with 12% lime, consistent with the strength increases determined in this study.

The strengthening mechanism that increases soil strength in lime stabilization applications occurs in two ways. The first of these mechanisms is ion exchange, which has a short-term effect. In ion exchange, weakly bonded cations such as sodium (Na^+^) and potassium (K^+^) found on the surface of clay soils are replaced by calcium (Ca^+^) ions in the lime. As a result, interparticle bonds are strengthened, increasing the soil’s cohesion and thus its shear strength. The increase in shear strength of the uncured soil samples in the graph shown in Figure 9 was thought to occur in this way. The second mechanism is pozzolanic reactions, which have a long-term effect. Pozzolanic reactions are time dependent and, during this stage, lime reacts with silica (SiO_2_) and alumina (Al_2_O_3_) in the clay minerals. As a result, calcium silicate hydrate (C-S-H) and calcium aluminate hydrate (C-A-H) compounds are formed. Cementation occurring between soil particles significantly increases strength while reducing water permeability. Pozzolanic reactions are generally known to begin after 24 h, and this mechanism was thought to be responsible for the increase in shear strength seen in Figure 10 and Figure 11.

**Figure 12:**
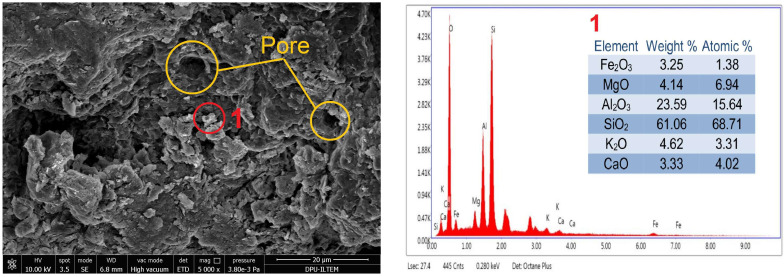
SEM-EDX analysis of 100% soil.

While the axial deformation percentage of the natural soil at the time of collapse was around 7%, this value gradually decreased to 2% and then to 1% with increased lime ratio in the mixture and the curing time. This result showed that the lime addition reduced the ductility of the material under load and caused it to exhibit brittle behavior. Studies on lime-stabilized samples in the literature also identified a shear failure mode similar to that observed in brittle materials [4,5]. After determining the UCS of the 28-day natural soil, the internal structure, surface morphology, and chemical composition of the sample were analyzed using SEM-EDX analysis. The 5000-fold magnified SEM image and analysis results are shown in Figure 12.

According to the EDX analysis of the 28-day-old natural soil, it was seen that it consisted of 61.06% SiO_2_ and 23.59% Al_2_O_3_ oxides by mass. There were also low amounts of 4.62% K_2_O, 4.14% MgO, and 3.25% Fe_2_O_3_, which made the soil red. When compared with the chemical composition of the natural soil given in Figure 4, it was seen that the principal oxides were similar. In addition, when the surface images shown in Figure 4 and Figure 12 were compared, it was seen that the microstructural properties, phase evolutions, and porosity changed significantly as a result of the chemical reactions that occurred as a result of compressing the sample at the optimum water content under a specific compaction energy and storing it under appropriate curing conditions for 28 days. While a compact internal structure was formed due to this change, there were also pore structures with sizes ranging from 2.9 to 5.7 μm.

### 3.4. Evaluation of the Mechanical Strength of Polypropylene Fiber-Reinforced Soil Mixtures

**Figure 13:**
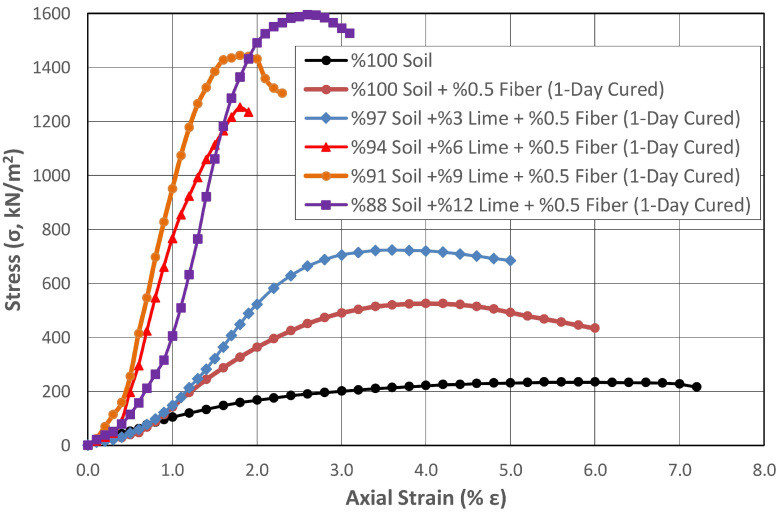
Change in the shear strength of the subgrade stabilized with different proportions of lime and 0.5% fiber after 1 day of curing.

**Figure 14:**
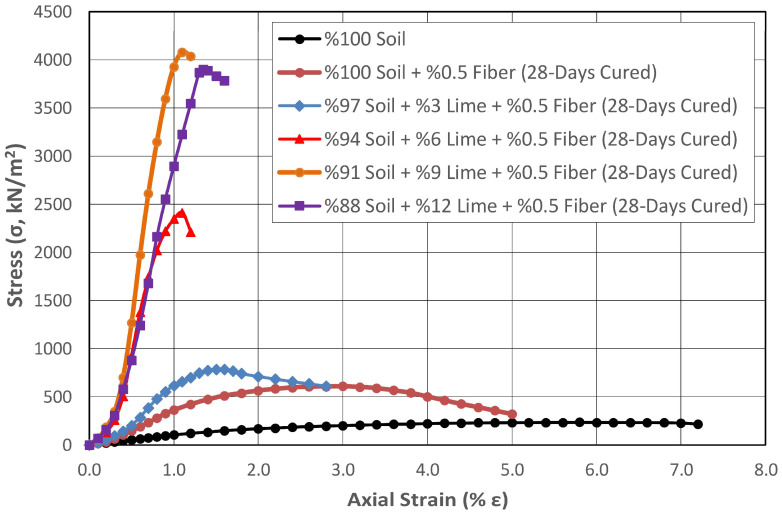
Change in the shear strength of the subgrade stabilized with different proportions of lime and 0.5% fiber after 28 days of curing.

High-plasticity subgrades exhibit shrinkage behavior depending on the change in water content, and they lose a significant amount of their strength at high water contents. Highway pavements located on such soils face serious risks. The use of polypropylene fibers and other stabilizing agents in improving the engineering properties of high-plasticity subgrades is becoming increasingly widespread due to the low price and easy availability of these materials. In addition to the fibers having high chemical and biological degradation resistance, they improve many engineering properties of the soils, especially the bearing capacity, since they are a material with high tensile strength. In this experimental study, synthetic fibers with a length of 12 mm at a rate of 0.5% by dry weight were mixed with natural and lime-stabilized subgrades, and the effect of fiber addition on the strength properties of these mixtures was investigated. To ensure homogeneous distribution of the synthetic fibers within the soil, before mixing with the soil, compressed air was applied in a closed chamber to separate the fibers from each other, which were then mixed with the soil in dry form. Figure 13 and Figure 14 illustrate the variation in shear strength of the subgrade stabilized with different lime contents and 0.5% fibers after curing periods of 1 day and 28 days, respectively.

While the strength of the natural soil increased by 124% when only 0.5% fiber was added and it was exposed to one day of curing, there was a 50% increase in strength due to fiber addition alone, independent of the curing effect. When only the fiber effect was examined in lime-added samples, regardless of the lime additive and curing, it was determined that the fibers increased the strength by 79% in samples containing 3% lime. Later, with growing lime ratios, the effect of fiber addition on strength decreased to 10% levels, and finally, this ratio decreased to 1.5% in the sample with a 12% lime addition ratio.

When the effect of lime addition at rates between 3% and 12% was examined in the one-day cured samples with fiber additive, the strength increased at rates between 38% and 204%, respectively.

It was observed that the increase in shear strength of the natural soil with 0.5% fiber addition and 28 days of curing was 159%. When the results were examined independently of the curing effect, it was determined that adding only 0.5% fibers to the natural soil increased the strength by 87%. When the effect of fiber addition on strength in lime-added cured samples was examined, it was determined that fibers increased the strength by 25% in samples containing 3% lime, and that the effect of fiber addition on strength increased up to 74% with growing lime rates. Finally, this rate decreased to 3.3% in the sample with 12% lime addition.

**Figure 15:**
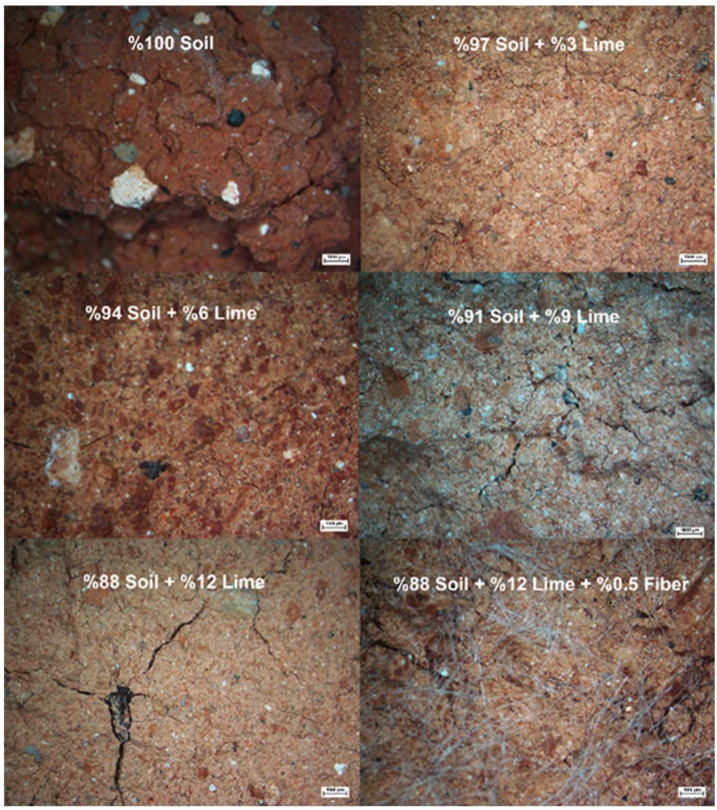
Images of shear planes in samples after unconfined compression tests were carried out.

Microscope photographs taken from the shear planes in the samples after unconfined compression tests were carried out on the natural soil and samples with different proportions of lime and fiber additives are presented in Figure 15.

The dark red appearance of the natural soil turned pink as the lime content in the mixture increased. It was observed that the lime was distributed homogeneously without clumping in the soil stabilized with different proportions of lime. In this way, chemical reactions between the soil and the homogeneously mixed lime contributed significantly to the increase in the compressive strength of the soil. Lime addition increased the strength and hardness of the soil by promoting the formation of stable calcium silicate hydrate (C-S-H) and calcium aluminate hydrate (C-A-H) gel products [36,37].

In addition, when the distribution of fiber additive in the soil was examined, it was seen that the fibers were distributed homogeneously in the soil in an irregular manner in all directions. The fact that the fibers were a material with high tensile strength and were located in different directions on the shear plane caused an increase in the resistance to collapse.

When the effect of fiber addition in chemical stabilization applications was evaluated in general, it was seen that fibers could increase the strength up to approximately 90% in low-strength mixtures. By contrast, the strength-increasing effect of fibers gradually decreased in high-strength samples.

Brittle fracture occurring in lime-stabilized soil causes a significant loss of strength after fracture [15,38]. The use of fibers increases the material’s ductility and prevents a rapid decrease in strength after the shear strength is exceeded.

### 3.5. Freeze–Thaw Durability Performance of Stabilized Soil Mixtures

Loss of stability of high-plasticity soils exposed to FTCs in cold climate regions is a significant engineering problem. At low temperatures, the freezing of water in the soil results in an increase in volume and swelling, and during thawing, an increase in the soil water content and softening of the soil occur. As a result of these cycles, as the soil loses strength, settlement under load increases, and damage occurs to the pavement.

**Figure 16:**
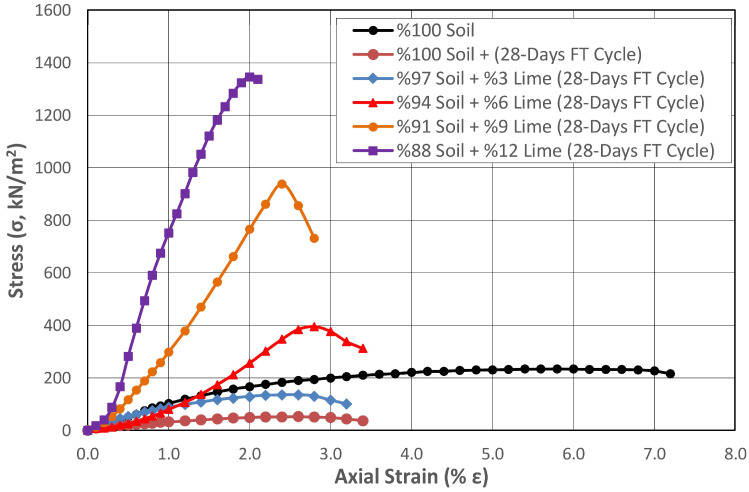
Change in the strength of the subgrade stabilized with different proportions of lime under the effect of FTCs.

**Figure 17:**
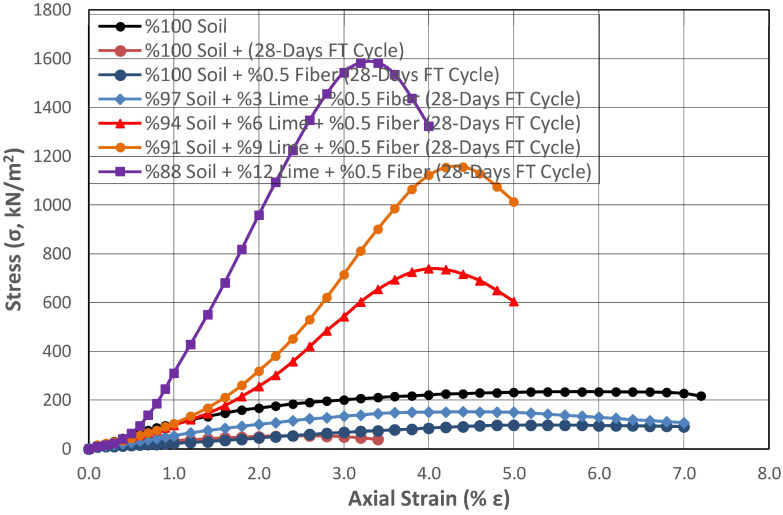
Change in the strength of the subgrade stabilized with different proportions of lime and 0.5% fiber under the effect of FTCs.

Therefore, the performance of natural subgrade mixtures stabilized with varying proportions of lime and synthetic fibers was investigated under the influence of FTCs. Regional climate conditions were considered in the FTCs, and the highest daytime temperature in winter was accepted as +10 °C, and the lowest nighttime temperature was accepted as −10 °C. The samples prepared for this purpose were wrapped in two layers of impermeable stretch film so that their water content would not change. They were frozen at −10 °C for 12 h in a programmable freezer with temperature and time adjustment, thawed at +10 °C for 12 h, and subjected to 24 h FTCs. Changes in the UCS of non-fibrous and fibrous soil samples stabilized with different amounts of lime subjected to 28-day FTCs were determined. The results obtained from this study are presented in Figure 16 and Figure 17, respectively.

It was determined that there was a considerable strength loss of 77% in the natural soil exposed to FTCs, without and with fiber additive. When the curing effect was taken into account, it was seen that this loss increased up to 84%. When the natural soil without and with fiber additive was stabilized with varying proportions of lime, the effect of FTCs on strength loss was reduced. In the soil stabilized with 12% lime, the highest lime mixture ratio was determined in this study, and the strength loss decreased to 64% and 59%, respectively.

The shear strength of the non-fibrous and fibrous soil stabilized with 3% lime, subjected to FTCs, was 155% and 186% higher than that of the natural soil, and 645% and 1290% higher in the soil stabilized with 6% lime, respectively. This ratio increased as the lime ratio in the mixture increased.

The results obtained from this study were consistent with those reported in the literature, demonstrating that the incorporation of lime and fibers into clayey soils effectively mitigates the strength reduction induced by FTCs. Wang et al. (2022) [39] examined the influence of FTCs on the UCS of lime-stabilized, basalt fiber-reinforced loess. They reported that the combined addition of basalt fibers and lime increased the soil strength and significantly improved its resistance to freeze–thaw damage. In a study in which Saygili and Dayan (2019) [40] examined the influence of incorporating silica fume and polypropylene fibers into lime-stabilized kaolinite clay on their strength and freeze–thaw performance, it was determined that the incorporation of these additives enhanced the compressive strength and improved the resistance to FTCs.

**Figure 18:**
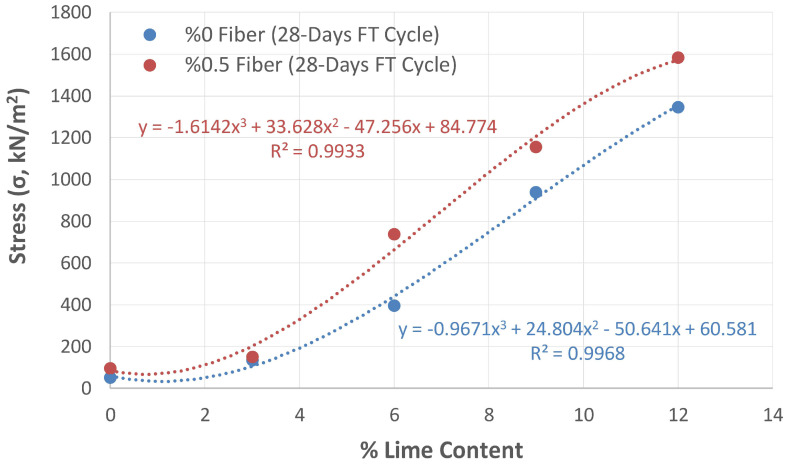
Change in the strength of the subgrade stabilized with different amounts of lime, both fibrous and non-fibrous, under the effect of FTCs.

The changes in the strength of the non-fibrous and fibrous mixtures stabilized with different proportions of lime exposed to FTCs in Figure 16 and Figure 17 are compared in Figure 18.

Figure 18 presents the graphs drawn according to the test results of the fibrous and non-fibrous samples. With the obtained equations, the percentage of lime (x value) to be used in stabilization to reach a particular strength (y value) can be determined. To compensate for the loss of strength caused by FTCs in synthetic fiber-free stabilization, it was calculated that 4.4% of the natural soil should be stabilized with lime when the shear strength is taken into account, and 5.1% of the natural soil should be stabilized when the 28-day cured strength is taken into account. These values were 3.3% and 4% in synthetic fiber stabilization, respectively. According to these results, if 0.5% synthetic fibers is used, a 25% saving can be achieved in the percentage of lime used in stabilization.

Stabilizing the subgrade with the lime percentage obtained using this method prevents the loss of strength caused by FTCs. In this way, it will be possible to protect the pavement from damage in the long term, as stability losses in the subgrade are minimized.

## 4. Conclusions

Because subgrade stability significantly impacts the long-term performance of road pavements, improving the engineering properties of weak subgrades is essential. Soil improvement methods generally aim to increase the soil bearing capacity, reduce settlement, and improve water resistance by reducing plasticity and volumetric change potential.

For this purpose, lime was mixed homogeneously with natural soil at different mixing ratios (3, 6, 9, and 12%) by dry mass, and the changes in compaction characteristics, such as consistency limits, optimum water content, and maximum dry unit weight, and engineering properties such as the UCS were investigated. In addition, 12 mm long polypropylene fibers were added to natural and lime-stabilized subgrade soils at 0.5%. The change in the shear strength of the subgrade soil exposed to different curing times and FTCs was then determined.

As the percentage of lime added to a high-plasticity subgrade increased, the liquid limit decreased by 4.7–25%, the plasticity index decreased by 4.9–38.1%, and the soil gradually departed from plastic behavior. Furthermore, when examining the effect of lime stabilization on the optimum water content and maximum dry unit weight values, it was observed that increasing the lime addition to the mixture up to 6% caused a 3% decrease in the optimum water content, while expanding the lime addition to 12% resulted in an increase in the optimum water content, reaching levels similar to those obtained for natural soil. Depending on the lime addition rate, the maximum dry unit weight value varied within a limited range, inversely proportional to the optimum water content.

When the strength characteristics of the subgrade soil and lime-stabilized mixtures were examined under uncured, 1-, and 28-day cured conditions, the shear strength of the soil stabilized with 3–12% lime increased by 56–63% under uncured conditions, while these increases were at the levels of 72–571% and 169–1511% under 1- and 28-day cured conditions, respectively. This result demonstrates the importance of preserving the sample for a sufficient curing period for pozzolanic reactions and strength gains in the lime stabilization method. Furthermore, the axial strain percentage of the lime-stabilized soil decreased significantly, indicating brittle behavior.

While 0.5% polypropylene fiber additive provided an increase of up to 90% in natural soil with lower strength, the higher strength of lime-stabilized mixtures reduced the strength-increasing effect of the fiber additive. As the fibers increased the strength by 79% in the soil stabilized with 3% lime, this increase decreased to 1.5% when the lime ratio in the mixture reached 12%. Mixing fibers with soil offers numerous benefits, including reduced soil brittleness, increased shear, compressive, and tensile strengths, and reduced swelling. The mechanical adhesion of fibers to the soil matrix positively affects deformation behavior and increases ductility. Furthermore, using fiber additive can reduce the amount of agent used in chemical stabilization, providing an environmentally friendly improvement.

When the effect of FTCs on the strength properties of the soil mixtures was examined, the strength loss caused by FTCs in natural soil with and without fiber additive reached up to 84%. Even though FTCs typically reduce the shear strength of soil samples, a significant increase in shear strength was observed in lime-stabilized samples compared to natural soil, attributed to pozzolanic reactions occurring under curing conditions. Although the strength loss caused by FTCs in the soil stabilized with 12% lime was 64%, the strength value of this soil was 2434% higher than the strength value of natural soil exposed to FTCs.

In cold climates, FTCs are the most critical factor affecting the long-term performance of road pavements. In subgrades stabilized with lime and synthetic fibers, strength losses caused by FTCs can be minimized, and the strength can even be increased. Furthermore, by improving the soil’s plasticity, volumetric changes resulting from changes in water content can be controlled, and long-term stability can be achieved.

## Figures and Tables

**Table 1:** Properties of high-plasticity subgrade.

Finer Sieve 4 %	Finer Sieve 200 %	Clay Content %	Atterberg Limits			Specific Gravity (Gs)	Classification of Soil	
			*W*L %	PL %	PI %		AASHTO	USCS
95.8	74.8	48.9	60.2	31.6	28.6	2.7	A-7-5	MH

**Table 2:** Chemical properties of high-plasticity subgrade.

SiO_2_ (%)	Al_2_O_3_ (%)	Fe_2_O_3_ (%)	CaO (%)	MgO (%)	K_2_O (%)	TiO_2_ (%)	Na_2_O (%)	MnO (%)	Others (%)
54.72	15.27	8.01	4.12	2.76	1.59	0.83	0.25	0.25	0.20

**Table 3:** Physical and chemical properties of slaked lime.

Physical Appearance	Ca(OH)_2_	Active CaO	CaO + MgO	Density (g/L)	>63 μ	>90 μ
Dry white powder	80–86%	60.6–65.15%	85–95%	375–500	7–10	3–6

**Table 4:** Characteristics of polypropylene (PP) fibers [29].

Appearance of Fibers	Natural White
Type	PP
Purity	%100 Pure
Specific Gravity	0.91 g/cm^3^
Length	12 (mm)
Profile/Diameter	Circular/18–20 µm
Tensile Strength	450–700 N/mm^2^ (Mpa)
Module of Elasticity	3000–3500 N/mm^2^ (Mpa)
Melting Point	162 °C
Ignition Point	593 °C
Acid, Salt, and Alkali Resistant	High
UV, Oxidation, Corrosion Resistance	High
Shelf-Life	Unlimited under Dry Conditions

**Table 5:** The impact of varying lime content on the plasticity characteristics of soil.

Mixing Ratios	*W*L	PL	PI
100% Soil	60.2	31.6	28.6
97% Soil + 3% Lime	57.4	30.2	27.2
94% Soil + 6% Lime	53.4	28.7	25.0
91% Soil + 9% Lime	49.8	28.2	21.6
88% Soil + 12% Lime	45.2	27.5	17.7

## Data Availability

All data are included in this article.

## References

[1] Wilkinson A., Haque A., Kodikara J., Adamson J., Christie D. (2010). Improvement of problematic soils by lime slurry pressure injection: Case study. J. Geotech. Geoenviron. Eng..

[2] Kavak A., Akyarlı A. (2007). A field application for lime stabilization. Environ. Geol..

[3] Castro-Fresno D., Movilla-Quesada D., Vega-Zamanillo Á., Calzada-Pérez M.A. (2011). Lime stabilization of bentonite sludge from tunnel boring. Appl. Clay Sci..

[4] Harichane K., Ghrici M., Missoum H. (2011). Influence of natural pozzolana and lime additives on the temporal variation of soil compaction and shear strength. Front. Earth Sci..

[5] Lin D.-F., Lin K.-L., Hung M.-J., Luo H.-L. (2007). Sludge ash/hydrated lime on the geotechnical properties of soft soil. J. Hazard. Mater..

[6] Sobhan K., Mashnad M. (2002). Tensile strength and toughness of soil-cement-fly-ash composite reinforced with recycled high-density polyethylene strips. J. Mater. Civ. Eng..

[7] Wang W., Lv B., Zhang C., Li N., Pu S. (2022). Mechanical characteristics of lime-treated subgrade soil improved by polypropylene fiber and class F fly ash. Polymers.

[8] Wang Z., Zhang W., Jiang P., Li C. (2022). The elastic modulus and damage stress-strain model of polypropylene fiber and nano clay modified lime treated soil under axial load. Polymers.

[9] Mohammed H.Z., Assefa E., Shantveerayya K. (2022). Utilization of sisal fiber as a subgrade soil reinforcement: A case study of Alem Tena Town along Modjo-Meki Expressway. Int. J. Mech. Eng..

[10] Puppala J., Musenda C. (2000). Effects of fiber reinforcement on strength and volume change behavior of expansive soils. Proceedings of the 79th Annual Meeting.

[11] Abd Al-Kaream K.W., Fattah M.Y., Hameedi M.K. (2022). Compressibility and strength development of soft soil by polypropylene fiber. Int. J. Geomate.

[12] Liu X., Han M., Liu T., Liu L. (2023). Macroscopic and microscopic characteristics of strength degradation of silty soil ımproved by regenerated polyester fibers under dry-wet cycling. Polymers.

[13] Khalid B., Alshawmar F. (2024). Comprehensive review of geotechnical engineering properties of recycled polyethylene terephthalate fibers and strips for soil stabilization. Polymers.

[14] Kumar A., Walia B., Mohan J. (2006). Compressive strength of fiber reinforced highly compressible clay. Constr. Build. Mater..

[15] Cai Y., Shi B., Ng C.W.W., Tang C. (2006). Effect of polypropylene fibre and lime admixture on engineering properties of clayey soil. Eng. Geol..

[16] Consoli N.C., Corte B.M., Festugato L. (2012). Key parameter for tensile and compressive strength of fiber-reinforced soil-lime mixtures. Geosynth. Int..

[17] Anggraini V., Asadi A., Huat K.B.B., Nahazanan H. (2015). Effects of coir fibers on tensile and compressive strength of lime-treated soft soil. Measurement.

[18] Moghal B.A.A., Chittoori B.C.S., Basha B.M. (2017). Effect of fiber reinforcement on CBR behavior of lime-blended expansive soils: Reliability approach. Road Mater. Pavement Des..

[19] Jackson N., Puccinelli J. (2006). Long-Term Pavement Performance (LTPP) Data Analysis Support: National Pooled Fund Study TPF-5(013)-Effects of Multiple Freeze Cycles and Deep Frost Penetration on Pavement Performance and Cost, FHWA-HRT-06-121.

[20] Guney Y., Aydilek A.H., Demirkan M.M. (2006). Geoenvironmental behavior of foundry sand amended mixtures for highway subbases. Waste Manag..

[21] Hotineanua A., Bouaskera M., Aldaooda A., Al-Mukhtar M. (2015). Effect of freeze–thaw cycling on the mechanical properties of lime-stabilized expansive clays. Cold Reg. Sci. Technol..

[22] Yarbaşı N., Kalkan E., Akbulut S. (2007). Modification of the geotechnical properties, as influenced by freze-thaw, of granular soils with waste additives. Cold Reg. Sci. Technol..

[23] Liu C., Lv Y., Yu X., Wu X. (2020). Effects of freeze-thaw cycles on the unconfined compressive strength of straw fiber-reinforced soil. Geotext. Geomembr..

[24] Ardakani A., Aliaghaei E. (2025). Dynamic response of carbon fiber reinforced clay under freeze-thaw cycles. Geotech. Geol. Eng..

[25] Boz A., Sezer A. (2018). Influence of fiber type and content on freeze-thaw resistance of fiber reinforced lime stabilized clay. Cold Reg. Sci. Technol..

[26] (2006). Standard Test Methods for Amount of Material in Soils Finer Than No. 200 (75-µm) Sieve.

[27] (2009). Standard Test Methods for Particle-Size Distribution (Gradation) of Soils Using Sieve Analysis.

[28] (2016). Standard Test Method for Particle-Size Distribution (Gradation) of Fine-Grained Soils Using the Sedimentation (Hydrometer) Analysis.

[29] Sengul T., Akray N., Vitosoglu Y. (2023). Investigating the effects of stabilization carried out using fly ash and polypropylene fiber on the properties of highway clay soils. Constr. Build. Mater..

[30] (2008). Standard Test Methods for Liquid Limit, Plastic Limit, and Plasticity Index of Soils.

[31] Harichane K., Ghrici M., Kenai S., Grine K. (2011). Use of natural pozzolana and lime for stabilization of cohesive soils. Geotech. Geol. Eng..

[32] (2008). Standard Test Methods for Laboratory Compaction Characteristics of Soil Using Standard Effort.

[33] McCarthy M.J., Csetenyi L.J., Sachdeva A., Dhir R.K. (2012). Identifying the role of fly ash properties for minimizing sulfate-heave in lime-stabilized soils. Fuel.

[34] (2008). Standard Test Method for Unconfined Compressive Strength of Cohesive Soil.

[35] Kavak A., Baykal A. (2012). Long-term behaviour of lime-stabilized kaolinite clay. Environ. Earth Sci..

[36] Chakraborty S., Nair S. (2017). Impact of different hydrated cementitious phases on moisture-induced damage in lime-stabilized subgrade soils. Road Mater. Pavement Des..

[37] Rajasekaran G., Rao N.S. (1996). Lime stabilization technique for the improvement of marine clay. Soils Found..

[38] Muntohar A.S., Widianti A., Hartono E., Diana W. (2013). Engineering properties of silty soil stabilized with lime and rice husk ash and reinforced with waste plastic fiber. J. Mater. Civ. Eng..

[39] Wang W., Guansen Cao G., Li Y., Zhou Y., Lu T., Zheng B., Geng W. (2022). Effects of freeze-thaw cycles on strength and wave velocity of lime-stabilized basalt fiber-reinforced loess. Polymers.

[40] Saygili A., Dayan M. (2019). Freeze-thaw behavior of lime stabilized clay reinforced with silica fume and synthetic fibers. Cold Reg. Sci. Technol..

